# Immunoproteomic analysis of outer membrane proteins and extracellular proteins of *Actinobacillus pleuropneumoniae *JL03 serotype 3

**DOI:** 10.1186/1471-2180-9-172

**Published:** 2009-08-20

**Authors:** Yonghong Liao, Junhua Deng, Anding Zhang, Mingguang Zhou, Yong Hu, Huanchun Chen, Meilin Jin

**Affiliations:** 1 Unit of Animal Infectious Diseases, National Key Laboratory of Agricultural Microbiology, Huazhong Agricultural University, Hubei, PR China; 2 College of Veterinary Medicine, Huazhong Agricultural University, Hubei, PR China; 3 Institute of Animal Science and Veterinary Medicine, Hubei Academy of Agricultural Sciences, Hubei, PR China

## Abstract

**Background:**

*Actinobacillus pleuropneumoniae *is the causative agent of porcine contagious pleuropneumonia, a highly contagious respiratory infection in pigs, and all the 15 serotypes are able to cause disease. Current vaccines including subunit vaccines could not provide satisfactory protection against *A. pleuropneumoniae*. In this study, the immunoproteomic approach was applied to the analysis of extracellular and outer membrane proteins of *A. pleuropneumoniae *JL03 serotype 3 for the identification of novel immunogenic proteins for *A. pleuropneumoniae*.

**Results:**

A total of 30 immunogenic proteins were identified from outer membrane and extracellular proteins of JL03 serotype 3, of which 6 were known antigens and 24 were novel immunogenic proteins for *A. pleuropneumoniae*.

**Conclusion:**

These data provide information about novel immunogenic proteins for *A. pleuropneumoniae *serotype 3, and are expected to aid in development of novel vaccines against *A. pleuropneumoniae*.

## Background

*Actinobacillus pleuropneumoniae*, a gram negative capsulated rod bacterium, is the etiologic agent of a severe, highly infectious and often fatal pleuropneumonia in swine, which is distributed world wide and results in severe losses in the swine industry. Based on capsular antigens, 15 serotypes of *A. pleuropneumoniae *to date have been documented, and all serotypes are capable of causing disease though differences in virulence have been described [1]. Among these serotypes, serotype 3 is one of the predominant serotypes in China [2].

So far, satisfactory protection has not been achieved in the *A. pleuropneumoniae *vaccination field in spite of intensive attempts made on inactivated whole-cell vaccines, live avirulent vaccines, which showed partial protection against challenges with homologous or heterologous serotypes[3]. Although currently available subunit vaccines contain important antigens, such as ApxI, ApxII and ApxIII, produced in various combinations by the different serotypes of *A. pleuropneumoniae*[4], they could not provide complete protection against *A. pleuropneumoniae*[3]. Thus identifying more conserved antigens is necessary for the development of novel vaccines, and in this study the immunogenic proteins of JL03 serotype 3 will be investigated to provide data for novel vaccine development. Extracellular proteins (ECPs) and OMPs in pathogens are involved in colonization, adhesion to and invasion of host cells. They interact directly with the host immune systems while playing crucial roles in the course of infections. Thus it is feasible to identify the important vaccine candidates from these sub-fractions. Currently, the immunoproteomic approach is a powerful tool to systematically identify immunogenic proteins from pathogens, and novel antigens have been successfully discovered from *S. streptococcus *[5], *B. anthrax *[6] and *S. flexneri *[7] by this approach from bacterial subfractions, such as outer membrane proteins.

Recently, Chung *et al*. performed systematically proteomic analysis on OMPs of *A. pleuropneumoniae *serotype 5b, and 47 OMPs were identified[8], and there have been attempts – but they are not recent and, therefore, could not use a proteomics approach. And no attempt has been reported so far in analysis of the ECPs of *A. pleuropneumonae*. The complete genome sequence of *A. pleuropneumonia *JL03 provided an essential database for applying immunoproteomic approach to JL03. In the present study, we report this approach to JL03 for the first time which involved the identification of immunogenic proteins from its OMPs and ECPs.

## Results and Discussion

### 2-DE profile of the ECPs and OMPs, immunoblotting analysis and identification of immunogenic proteins

**Figure 1:**
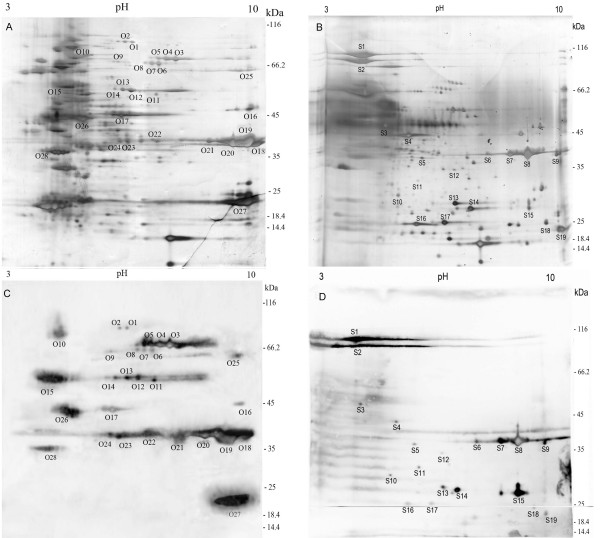
**2-DE profile of ECPs and OMPs and immunoblot**. 2-DE profile of OMPs (A) and ECPs (B) from *A. pleuropneumoniae *JL03 strain. Preparative gel stained with Silver Nitrate. Immunoblot of OMPs and ECPs from convalescent sera (C) and (D). The gel spots were encoded using a letter followed by the protein number, which was assigned based on their similar locations on different gels/membranes.

In the present study, linear immobilized pH gradient strips (3–10 L IPG 13 cm) and 10% SDS-PAGE gels were used for the prepared samples separation. Figure 1A and 1B show the 2-DE profile of OMPs and ECPs of *A. pleuropneumoniae *JL03. The 2-DE and immunoblotting were repeated three times and the results were reproducible. A total of 110 spots and 98 spots were detected on the silver-stained gels of OMPs and ECPs respectively by the software ImageMaster v 6.01. After immunoblotting analysis with convalescent sera, 28 immunoreactive spots from OMPs (Figure 1A and 1C) were identified, and they represented 17 proteins. Chung *et al*. recently identified 47 OM proteins from *A. pleuropneumoniae *5b with an optimized extraction protocol based on the sucrose-density gradient which yielded preparations highly enriched for OM proteins and lipoproteins[8], and 10 of the 47 OM proteins were identified as immunogenic proteins in this study. In addition, Rhonda *et al*. recently demonstrated the sucrose-density gradient extraction of outer membranes in *Campylobacter jejuni *produced purer sample than carbonate extraction [9] that was applied in this study. So further study needs to be tried on immunoproteomic analysis of other serotypes of *A. pleuropneumoniae *with the optimized OMP extraction protocol of Chung *et al*. for search of more immunogenic OMPs. All the 19 immunoreactive spots from ECPs (Figure 1B and 1D) that represented 16 proteins were identified whereas no specific immunoreactive protein spot was observed from OMPs and ECPs using control sera. The detailed Peptide Mass Fingerprinting (PMF) results of the immunoreactive proteins are listed in supplemental table S1 [see additional file 1]. Overall, values of gel estimated *pI *and MW are matched well with their theoretical ones but some discrepancies still exist. Similar migration for several proteins has been observed in proteomic analysis of other pathogens previously[10,11]. This might be due to the presence of natural isoforms, posttranslational processing, and/or modification, or an artifact caused by sample preparation.

All identified proteins were predicted by PSORTb 2.0, 10 proteins are annotated as periplasmic proteins, 7 are OMPs, 2 are extracellular proteins, 2 are cytoplasmic proteins, 1 is cytoplasmic membrane protein, and 8 are unknown. The detailed functions of the identified immunoreactive proteins are shown in supplemental table S1 [see additional file 1] according to the results predicted by COGnitor.

Interestingly, 3 immunogenic proteins, MomP1, MomP2 and elongation factor Tu were identified from OMPs and ECPs simultaneously, which might be due to outer membrane vesicles released in the milieu [12], from which outer membrane proteins have been identified successfully from *E. coli *and *A. pleuropneumoniae*[8,13], and to dual localization of elongation factor Tu [14].

### Characterization of identified immunogenic proteins

Our immunogenic approach led to the identification of 6 known antigens of *A. pleuropneumoniae*, namely MomP1, MomP2, ApxIIA, ApxIIIA, Na+-translocating NADH-ubiquinone oxidoreductase subunit A (NqrA) and outer membrane ferric hydroxamate receptor (FhuA)[15,16]. And other well-known antigens, like ApxI, ApxIV, outer membrane lipoprotein A (OmlA), outer membrane protein precursor (PalA) and Transferrin binding proteins (Tbp) proteins could not be detected in the present study. ApxIV is only induced *in vivo *and JL03, serotype 3 strain, can not produce ApxI, and therefore we could not detect ApxI and ApxIV. Tbp proteins are expressed under iron limited conditions and the cells we collected were not prepared under such conditions. So Tbp proteins did not appear in our results. The highly hydrophobic nature of OmlA and PalA might cause their loss during extraction procedure. PalA has been proved to be detrimental when used in vaccines[17], and thus we should be cautious about similar immunogenic proteins while applying them to vaccine development.

In addition, we found 16 immunogenic proteins that had an significant sequence similarity to known proteins, and they have already been shown immunogenic in certain pathogenic bacteria, but not in *A. pleuropneumoniae *before, namely D15/OmpD, LppB, FrdA, MDH, FepA, FrpB, TufB, PotD, GapA, ZnuA, TIG, DegP, TufB, PsaA, FkpA and PTA. The homolog D15/Omp85 is an essential component for outer membrane biogenesis and OMP assembly [18,19]. The immunogenicity of D15 and its homolog Omp87 has been demonstrated in *Haemophilus ducreyi *[20] and *Pasteurella multocida *[21] respectively. Furthermore, antibodies against the COOH-terminal "surface antigen" domain of D15 are protective against *Haemophilus influenzae *infection in animal models [22]. The immunoreactive spot O16 was homologous to LppB and shared 49% sequence identity with LppB of *H. somni *that has been shown as an immunodominant protein [23], and the gene *lppB *of *A. pleuropneumoniae *is important for survival during infection[24]. Fumarate reductase (FrdA) was found to be involved in biosynthesis of flagella and cell motility, which might contribute to an significantly attenuated *A. pleuropneumoniae *[25] and loss of the ability in colonizing in the gastric mucosa in *Helicobacter pylori*[26] after *frdA *genes were inactivated. Furthermore, Joseph *et al*. described FrdA as an antigen in *Brucella abortus *[27]. FepA, FrpB and HbpA are important components in several ABC transport pathways for obtaining iron or regulating iron utilization *in vivo *or *vitro*. The immunogenic activity of FepA and FrpB was shown in *Klebsiella pneumoniae *[28] and *Neisseria meningitides *[29] respectively, and HbpA was widely conserved and served as an antigen in *Leptospira interrogans*[30]. Moreover, homologous analysis of these proteins at NCBI revealed a high level identity (>98%) with the sequenced serotype 1, 5 and 7 strains respectively. These suggest that they might be new common antigens for *A. pleuropneumoniae*. High-affinity zinc uptake system protein ZnuA precursor, was essential of *B. abortus *for intracellular survival and virulence in mice[31] and shown immunogenic in *Streptococcus suis*[5]. PsaA is needed for the adherence of pneumococcal cells and antibodies to PsaA contributed to reduce the nasopharyngeal colonization of challenged pneumococcal cells [32,33]. DegPs, a member of the widely conserved HtrA family of serine proteases, were frequently identified as antigens in other pathogens, such as *B. abortus *[34] and *Chlamydia trachomatis *[35]. Besides, trigger factor (TIG) has been demonstrated to be an excellent candidate for vaccination against *Brucella melitensis *[36] and a virulence-related protein in *Listeria monocytogenes *[37], and similar findings were described about malate dehydrogenase (MDH) of *Candida albicans *[38] and spermidine/putrescine-binding periplasmic protein (PotD) of *Streptococcus pneumoniae *[39]. Glyceraldehyde 3-phosphate dehydrogenase (GapA) has been proven to be antigenically conserved proteins, suggesting potential for vaccines in several microorganisms [40]. Homologous protein of translation elongation factor EF-Tu (TufB), a very abundant protein, had been detected in immunological researches of other bacteria, such as *C. trachomatis*[41] and *Shigella flexneri*[7]. The periplasm of gram-negative bacteria is well equipped with ATP-independent chaperones and folding catalysts, including peptidyl-prolyl isomerases (FkpA). It is reported recently that FkpA was found to be immunogenic in *Bordetella pertussis*[42]. Phosphate acetyltransferase (PTA), an enzyme that catalyzes the reversible transfer of the acetyl group from acetyl phosphate to coenzyme A plays a major role in the energy-yielding metabolism[43] and recently has been reported to be immunogenic in *S. suis*[5].

It is notable that CbiK, IlvG, FepB, AfuC, FatB, GGBP, CysG and Ttg2D, are reported to be immunogenic proteins for the first time in this study whose functions have been biologically demonstrated in some bacteria. Putative periplasmic binding protein CbiK is involved in the uptake of Ni^2+^, a cofactor required for urease activity that is important in pathogenesis of pleuropneumonia [44]. The *ilv *gene of *Brucella suis *has been identified as a virulence gene[45], and its product, acetohydroxyacid synthase, catalyzes the first common step in the biosynthetic pathway of the branched-amino acids such as leucine, isoleucine, and valine. Iron is essential for bacterial growth, especially for *A. pleuropneumoniae *in invading and reproducing in porcine respiratory tract where iron is limited. Iron-restriction is an important signal that regulates expression of many genes including some coding for virulence factors[46]. FepB, AfuC and FatB are components of known iron transport pathways, and the immunogenic reactivity of these proteins in this study indicates that these iron-uptake proteins might be potential candidates for development of subunit vaccines. D-Galactose/D-glucose binding protein (GGBP) is a bacterial periplasmic protein, an initial component for both chemotaxis towards galactose and glucose and active transport of the two sugars in *Escherichia coli*[47]. The crystal structure of uroporphyrinogen-III methylase (CysG) from *Thermus thermophilus *has been reported[48] and the *cysG *gene of *Salmonella typhimurium *is involved in synthesis of both cobalamin (B12) and siroheme[49]. The ttg2D gene encodes a periplasmic component of an ABC-type transport system related to resistance to organic solvents, and Ttg proteins of *Pseudomonas putida *and *N. meningitidis *were verified to participate in the uptake of L-glutamate[50].

Novel vaccine candidates need to be highly conserved between strains and so that they induce cross-protection against *A. pleuropneumoniae*. Recently Goure *et al*. have identified *A. pleuropneumoniae *genes that are conserved among all 15 serotypes by comparative genomic hybridization[51]. Of these conserved genes, the genes encoding proteins MomP1 (OMP P5), MomP2 (OMP P5), D15 (OmpD), LppB, PotD, FkbP and FrpB were observed in our results. Besides, NqrA has been demonstrated to be common to all serotypes[15]. Thus these conserved proteins could potentially induce protection against a wide variety of strains and are attractive vaccine candidates.

## Conclusion

In conclusion, the 2DE in combination with Western blot is a specific and powerful method to discover novel antigens from bacterial pathogens. In this study, the identified immunogenic proteins from ECPs and OMPs may be significant for the development of new efficient vaccine against *A. pleuropneumoniae*. The protective efficacy of the identified immunogenic proteins either by alone or in different combinations remains to be evaluated in further studies. The data of this study are expected to aid in development of novel vaccines against *A. pleuropneumoniae*. The present study has focused on 2DE analysis coupled with Western blotting.

## Methods

### Bacterial strains and culture conditions

*A. pleuropneumoniae *JL03 [52], a Chinese field isolate strain of serotype 3, was used for this study. Bacteria were routinely maintained on tryptic soy agar (Difco Laboratories, Detroit) containing 10% bovine calf serum and 0.01% nicotinamide adenine dinucleotide (NAD) and cultured in brain heart infusion (BHI, Difco Laboratories, Detroit) media containing 0.01% NAD in a rotary incubation shaker running at 180 rev min. For protein extractions, overnight bacterial cultures were diluted 1:100 in 1 L BHI broth and grown aerobically at 37°C at 180 rev min. Three independent cultures at different days were prepared for ECPs and OMPs preparation respectively.

### Extracellular proteins sample preparation

Growth was stopped at the early exponentional phase at an OD_600 _of ~0.4. Supernatant containing protease inhibitor cocktail (Roche, Mannheim, Germany) was collected by centrifugation for 10 min at 7200 × g (Sigma 3K12, Nr. 12150) at 4°C and filtered with 0.22 μm membrane (Millipore, USA). The supernatant was then treated with 15% TCA (Sigma Chemical) in ice for 30 min to precipitate protein. The precipitate was collected by centrifugation at 10 000 × g for 20 min at 4°C. Then the precipitated protein was washed three times with ice cold acetone containing 0.1% DTT to remove traces of TCA, and acetone was finally removed by speed vacuum treatment. Extracellular proteins were solubilized with 7 M urea, 2 M thiourea, 4% CHAPS, and 65 mM DTT. Protein concentration was determined by the GE Healthcare 2-D Quant Kit (Piscataway, NJ, USA).

### OMPs sample Preparation

OMPs were prepared according to Molloy *et al *[53], with minor modifications. Briefly, cells were harvested in the middle exponential phase (OD_600_, ~1.0) by centrifugation for 10 min at 7200 × g (Sigma 3K12, Nr. 12150) at 4°C, and then the pellets were washed twice with cold 50 mM Tris-EDTA (pH 7.5) containing protease inhibitor cocktail (Roche, Germany) and subsequently the cells were suspended in 10 ml 50 mM Tris-EDTA (pH 7.5) and ruptured by sonication at 50 watts; pulse on, 3 sec.; pulse off, 3 sec. (Sonorex Digital 10 P Sonicator Bandelin, Berlin, Germany), till the value of OD_600 _decreased to 1/10 of its origin. Unbroken cells and cellular debris were removed by centrifugation at 6000 × g for 10 min. The supernatant was diluted 10-fold with ice cold 0.1 M Na_2_CO_3 _(pH 11), and stirred slowly on ice for 1 h. The OMPs were pelleted by ultracentrifugation in a Beckman Optima MAX ultracentrifuge (Palo Alto, CA, USA) at 100 000 × g for 1 h at 4°C. The supernatant was then discarded, and the pellet was resuspended and washed in 50 mM Tris-EDTA (pH 7.5) twice. The pellet was collected by centrifugation at 100 000 × g for 1 h at 4°C and subsequently solubilized in a lysis buffer (7 M urea, 2 M thiourea, 4% CHAPS, 1% Triton X-100 and 65 mM DTT). Protein concentration was determined by the GE Healthcare 2-D Quant Kit.

### Preparation of convalescent sera

Bacteria JL03 were prepared as described above and the cells were washed once with PBS before dilution. Five pigs free of *A. pleuropneumoniae *were inoculated intratracheally at dose of 1.0 × 10^7^CFU/pig in PBS to prepare the convalescent sera, and three pigs survived. Twenty days after the first infection, the survivors were rechallenged with another identical dose of JL03. Sera were collected a week after the second inoculation and evaluated. Antibody titer of mixed sera from survivors 1:512 was measured by IHA kit (Lanzhou Bioproducts Factory, Lanzhou, China). Sera were collected before inoculation as control sera. All animals were housed and maintained in isolation facilities in accordance with the **Animal Care and Use Committee guidelines of Huazhong Agricultural University**.

### 2-DE and immunoblotting analysis

IEF was performed with the IPGphor II™ system (GE Healthcare, USA) and the Immobiline DryStrip™ IPGstrips of 13 cm (linear 3–10 pH gradient) according to Gorg *et al*[54]. The prepared ECPs and OMPs (150 μg/strip) was mixed with rehydration buffer (7 M urea, 2 M thiourea, 2% w/v CHAPS, 1%w/v DTT, 0.5%v/v IPG buffer, 0.002% w/v bromophenol blue). The ECPs and OMPs samples were focused for 50 kVh and 75 kVh respectively. After IEF, three gels were run as follows. The IPGstrips were respectively equilibrated for 15 min with 10 mg/ml DTT and 40 mg/ml iodoacetamide in equilibration buffer (6 M urea, 2% w/v SDS, 30% v/v glycerol, 0.002% w/v bromophenol blue, 50 mM Tris-HCl, pH 8.8). After equilibration, the second dimension electrophoresis was performed on a 10% SDS polyacrylamide gel using Hoefer SE600 Ruby (Amersham Biosciences). Proteins of one gel were visualized by staining with silver nitrate (Bio Basic Inc). And gel evaluation and data analysis were carried out using the ImageMaster v 6.01 program (GE Healthcare, USA).

Immunoblot was performed according to Mansfield [55]. Gels were blotted onto PVDF transfer membranes (Hybond-P, 0.45 mm; Amersham Biosciences). The membranes were blocked in 5% BSA in TBS +0.05%(v/v) Tween 20 for 1 h at room temperature and probed with the convalescent swine sera and control sera (1:1000), for 1 h at room temperature, and then were washed and incubated with goat anti-porcine IgG (H+L) -HRP (1:5,000) (Southern Biotech, Birmingham, AL, USA) for 1 h at room temperature, followed by development with Supersignal west pico chemiluminescent substrate (Pierce, Rockford, IL, USA) and imaged on the Image Station 2000 MM (Kodak, Rochester, NY, USA). All experiments were done in triplicate.

### In-gel digestion of proteins[5]

Protein spots of interest were excised from gels and detained with 100 μl 30 mM potassium ferricyanide and 100 μl 100 mM sodium thiosulfate (at a ratio of 1:1). And the gel pieces were shrunken with 50 μl acetonitrile and then re-swollen with 5 μl of 25 mM ammonium bicarbonate containing 10 ng of trypsin at 4°C for 30 min. In-gel tryptic degradation was performed overnight at 37°C, followed by three subsequent extractions. The pooled extracts were lyophilized and reconstituted in 2 μl 0.1% TFA v/v prior to MALDI-TOF MS analysis.

### MALDI-TOF MS analysis and database searchs

The sample solution with equivalent matrix solution was applied onto the MALDI-TOF target and prepared for MALDI-TOF-MS analysis according to a previously described procedure [56]. CHCA was used as the matrix. MALDI-TOF spectra were calibrated using trypsin autodigestive peptide signals and matrix ion signals. MALDI analysis was performed by a fuzzy logic feedback control system (Ultraflex αMALDI TOF/TOF system Bruker, Karlsruhe, Germany) equipped with delayed ion extraction. PMF data were searched against the database of JL03 by MASCOT licensed in-house and the NCBInr database using the MASCOT program http://www.matrixscience.com.

### Bioinformatics tools

COGnitor http://www.ncbi.nlm.nih.gov/COG/old/xognitor was applied to sort the identified proteins of *A. pleuropneumoniae *JL03 into functional categories. PSORTb v.2.0 is accessible at http://www.psort.org/psortb/index.html and applied to predict the subcellular location of the identified proteins.

## Authors' contributions

YL and MJ carried out the molecular genetic studies, participated in the sequence alignment and drafted the manuscript. AZ, JD, YH, YL and MZ carried out the immunoassays. MZ and JD participated in the sequence alignment. MJ and YL participated in the design of the study and performed the statistical analysis. HC conceived of the study, and participated in its design and coordination. All authors read and approved the final manuscript.

## Supplementary Material

Additional file 1 **Supplementary table S1**. List of immunoreactive proteins of OMPs and ECPs Click here for file
