# Supplementary material for: Immunoproteomic analysis of outer membrane proteins and extracellular proteins of Actinobacillus pleuropneumoniae JL03 serotype 3
Source: BMC Microbiol. 2009 Aug 20;9:172. doi: 10.1186/1471-2180-9-172 (PMC2741471; doi:10.1186/1471-2180-9-172)
Supplement: Additional file 1 — Supplementary table S1. List of immunoreactive proteins of OMPs and ECPs [file 1471-2180-9-172-S1.doc]

## Supplemental Table 1: List of immunoreactive proteins of OMPs and ECPs

| Identification Protein | Spot No. | Acc. ID  NCBInr | Gene ID  NCBI | Theoretical p*I/*MW^a)^ | MOWS^b)^ E score | peptide match(sequence coverage, %)^C)^ | Gene symbol | PSORTb  localization | PSORTb  Probability^d)^ | Function |
| --- | --- | --- | --- | --- | --- | --- | --- | --- | --- | --- |
| **outer membrane protein P5 precursor** | **S6** | **165976856** | **5850797** | **8.75/39679** | **172** | **23 (70%)** | **momP1^e)^** | **OuterMembrane** | **9.93** | **Cell envelope biogenesis** |
| **outer membrane protein P5 precursor** | **S7** | **165976856** | **5850797** | **8.75/39679** | **67** | **10 (35%)** | **momP1^e)^** | **OuterMembrane** | **9.93** | **Cell envelope biogenesis** |
| **outer membrane protein P5 precursor** | **S8** | **165976856** | **5850797** | **8.75/39679** | **103** | **18 (51%)** | **momP1^e)^** | **OuterMembrane** | **9.93** | **Cell envelope biogenesis** |
| **outer membrane protein P5 precursor** | **S15** | **165976856** | **5850797** | **8.75/39679** | **80** | **14 (51%)** | **momP1^e)^** | **OuterMembrane** | **9.93** | **Cell envelope biogenesis** |
| **outer membrane protein P5 precursor** | **O19** | **165976856** | **5850797** | **8.75/39679** | **191** | **22 (74%)** | **momP1^e)^** | **OuterMembrane** | **9.93** | **Cell envelope biogenesis** |
| **outer membrane protein P5 precursor** | **O20** | **165976856** | **5850797** | **8.75/39679** | **189** | **22 (85%)** | **momP1^e)^** | **OuterMembrane** | **9.93** | **Cell envelope biogenesis** |
| **outer membrane protein P5 precursor** | **O23** | **165976856** | **5850797** | **8.75/39679** | **202** | **23(84%)** | **momP1^e)^** | **OuterMembrane** | **9.93** | **Cell envelope biogenesis** |
| **outer membrane protein P5 precursor** | **O24** | **165976856** | **5850797** | **8.75/39679** | **191** | **22 (74%)** | **momP1^e)^** | **OuterMembrane** | **9.93** | **Cell envelope biogenesis** |
| **outer membrane protein P5 precursor** | **O27** | **165976856** | **5850797** | **8.75/39679** | **147** | **21 (76%)** | **momP1^e)^** | **OuterMembrane** | **9.93** | **Cell envelope biogenesis** |
| **outer membrane protein P5 precursor** | **S9** | **165977295** | **5851926** | **9.51/38692** | **110** | **17 (49%)** | **momP2^e)^** | **OuterMembrane** | **9.93** | **Cell envelope biogenesis** |
| **outer membrane protein P5 precursor** | **O18** | **165977295** | **5851926** | **9.51/38806** | **50** | **13 (30%)** | **momP2^e)^** | **OuterMembrane** | **9.93** | **Cell envelope biogenesis** |
| elongation factor Tu | O26 | 165976941 | 5851358 | 5.28/43553 | 140 | 20 (63%) | tufB^e)^ | Cytoplasmic | 9.26 | Translation, ribosomal structure and biogenesis |
| elongation factor Tu | S4 | 165976941 | 5851358 | 5.28/43553 | 164 | 22 (67%) | tufB^e)^ | Cytoplasmic | 9.26 | Translation, ribosomal structure and biogenesis |
| RTX-III toxin determinant A | S1 | 165976753 | 5852555 | 5.62/112684 | 140 | 30 (35%) | apxIIIA | Extracellular | 10 | Secondary metabolites biosynthesis, transport and catabolism |
| hemolysin A | S2 | 165976376 | 5852343 | 5.45/102501 | 130 | 27 (31%) | apxIIA | Extracellular | 10 | Secondary metabolites biosynthesis, transport and catabolism |
| **Na+-translocating NADH-ubiquinone oxidoreductase subunit A** | **O17** | **165975606** | **5851670** | **5.91/48771** | **154** | **22 (55%)** | **nqrA**^e)^ | **Unknown** |  | **Energy production and conversion** |
| **outer membrane ferric hydroxamate receptor** | **O6** | **165977462** | **5851905** | **6.93/77123** | **242** | **31 (50%)** | **fhuA**^e,f)^ | **OuterMembrane** | **9.93** | **Inorganic ion transport and metabolism** |
| **outer membrane ferric hydroxamate receptor** | **O7** | **165977462** | **5851905** | **6.93/77123** | **135** | **24 (45%)** | **fhuA**^e,f)^ | **OuterMembrane** | **9.93** | **Inorganic ion transport and metabolism** |
| **outer membrane ferric hydroxamate receptor** | **O8** | **165977462** | **5851905** | **6.93/77123** | **115** | **20 (37%)** | **fhuA**^e,f)^ | **OuterMembrane** | **9.93** | **Inorganic ion transport and metabolism** |
| **outer membrane protein D-15** | **O1** | **165975871** | **5852202** | **6.49/89233** | **56** | **18 (29%)** | **ompD**^e)^ | **OuterMembrane** | **9.93** | **Cell envelope biogenesis** |
| **outer membrane protein D-15** | **O2** | **165975871** | **5852202** | **6.49/89233** | **82** | **20 (34%)** | **ompD**^e)^ | **OuterMembrane** | **9.93** | **Cell envelope biogenesis** |
| **outer membrane antigenic lipoprotein B** | **O16** | **165977377** | **5851473** | **9.08/41677** | **56** | **13 (36%)** | **lppB**^e)^ | **OuterMembrane** |  | **Cell envelope biogenesis** |
| fumarate reductase flavoprotein subunit | O9 | 165976959 | 5850804 | 5.95/66466 | 91 | 20 (38%) | frdA^e)^ | Periplasmic | 9.44 | Energy production and conversion |
| iron-regulated outer membrane protein | O3 | 165976719 | 5852337 | 7.74/77154 | 191 | 28 (49%) | fepA^e,f)^ | OuterMembrane | 9.93 | Inorganic ion transport and metabolism |
| iron-regulated outer membrane protein | O4 | 165976719 | 5852337 | 7.74/77154 | 219 | 32 (54%) | fepA^e,f)^ | OuterMembrane | 9.93 | Inorganic ion transport and metabolism |
| iron-regulated outer membrane protein | O5 | 165976719 | 5852337 | 7.74/77154 | 145 | 22 (49%) | fepA^e,f)^ | OuterMembrane | 9.93 | Inorganic ion transport and metabolism |
| **iron-regulated outer membrane protein** | **O25** | **165975732** | **5851335** | **9.56/73081** | **77** | **23 (31%)** | **frpB**^e,f)^ | **OuterMembrane** | **9.93** | **Inorganic ion transport and metabolism** |
| heme-binding protein A | O12 | 165977456 | 5851924 | 6.74/60563 | 140 | 23 (55%) | hbpA^e,f)^ | Periplasmic | 9.94 | Inorganic ion transport and metabolism |
| heme-binding protein A | O13 | 165977456 | 5851924 | 6.74/60563 | 87 | 12 (32%) | hbpA^d,e)^ | Periplasmic | 9.94 | Amino acid transport and metabolism |
| heme-binding protein A | O14 | 165977456 | 5851924 | 6.74/60563 | 96 | 14 (37%) | hbpA^e,f)^ | Periplasmic | 9.94 | Amino acid transport and metabolism |
| periplasmic zinc transporter | S12 | 165976875 | 5850850 | 6.10/35810 | 60 | 13 (32%) | znuA^e,f)^ | Periplasmic | 9.76 | Inorganic ion transport and metabolism |
| ABC-type metal ion transport system, periplasmic component/surface adhesin | S13 | 190149543 | 6397088 | 6.78/32893 | 98 | 12 (55%) | psaA^e,f)^ | CytoplasmicMembrane | 8.6 | Inorganic ion transport and metabolism |
|
| periplasmic serine protease | S18 | 165976713 | 5852293 | 6.17/49169 | 56 | 11 (26%) | degP^e)^ | Periplasmic | 9.76 | Posttranslational modification, protein turnover, chaperones |
| trigger factor | S3 | 165976936 | 5851732 | 4.98/47994 | 136 | 21 (61%) | tig^e)^ | Unknown |  | Posttranslational modification, protein turnover, chaperones |
| malate dehydrogenase | S11 | 165976716 | 5852257 | 5.56/33555 | 60 | 11 (43%) | mdh^e)^ | Unknown |  | Energy production and conversion |
| **spermidine/putrescine-binding periplasmic protein** | **O28** | **165975828** | **5851376** | **5.22/39611** | **110** | **16 (66%)** | **potD**^e)^ | **Periplasmic** | **9.76** | **Amino acid transport and metabolism** |
| glyceraldehyde 3-phosphate dehydrogenase | O22 | 165975894 | 5851715 | 6.54/35862 | 71 | 13 (38%) | gapA^e)^ | Cytoplasmic | 9.26 | Carbohydrate transport and metabolism |
| **FKBP-type peptidyl-prolyl cis-trans isomerase** | **S16** | **165977076** | **5850789** | **6.66/26053** | **60** | **13 (46%)** | **fkpA**^e)^ | **Periplasmic** | **9.76** | **Posttranslational modification, protein turnover, chaperones** |
| phosphate acetyltransferase | O10 | 165976056 | 5852774 | 5.50/77017 | 47 | 13 (26%) | pta^e)^ | Unknown |  | Energy production and conversion |
| **putative periplasmic binding protein CbiK** | **S17** | **165977060** | **5852843** | **6.61/25572** | **92** | **11 (43%)** | **cbiK**^e,f)^ | **Unknown** |  | **unknown** |
| acetohydroxy acid synthase II large subunit | O15 | 165975556 | 5851476 | 5.27/59120 | 59 | 15 (36%) | ilvG^e)^ | Unknown |  | Amino acid transport and metabolism |
| ABC-type Fe3+-hydroxamate transport system,periplasmic component | S5 | 165977228 | 5851463 | 6.09/41250 | 77 | 16 (38%) | fepB^e,f)^ | Periplasmic | 9.76 | Inorganic ion transport and metabolism |
| ferric transport protein | O21 | 165976825 | 5851100 | 6.26/39726 | 107 | 16 (50%) | afuC^e,f)^ | Unknown |  | Inorganic ion transport and metabolism |
| ABC-type enterochelin transport system,periplasmic component | S14 | 165976130 | 5852677 | 7.85/32490 | 107 | 15 (74%) | fatB^e,f)^ | Periplasmic | 9.76 | Inorganic ion transport and metabolism |
| periplasmic sugar-binding protein | S10 | 165976855 | 5851177 | 5.66/35418 | 70 | 13 (50%) | GGBP^e)^ | Periplasmic | 9.76 | Carbohydrate transport and metabolism |
| uroporphyrinogen-III methylase | O11 | 165977287 | 5851879 | 7.21/53176 | 42 | 12 (34%) | cysG | Unknown |  | Coenzyme metabolism |
| ABC-type transport system involved in resistance to organic solvents, auxiliary component | S19 | 165976709 | 5852698 | 9.65/23399 | 52 | 14 (52%) | ttg2D^e)^ | Unknown |  | Cell motility and secretion |

Bold text denotes the immunogenic proteins were also identified as OM proteins by Chung *et al.* 2007 in Proteomics.

a) Theoretical molecular weight and iso-electric point was determined using ExPASy compute MW/pI tool (http://us.expasy.org/tools/pi tool.html).

b) Mowse score is the score based on mowse algorithm when the protein identification was performed by peptide mass fingerprinting (matrix-assisted laser desorption ionization-mass spectometry MALDI-MS]) with the search program Mascot ([www.matrixscience.com](http://www.matrixscience.com/)).

c) Mass spectrometry results. Proteins were identified by MALDI-TOF analysis. The number of peptides matched and the sequence coverage of matched peptides is given.

d) Localization predictions based on PSORTb evaluation. The values are localization probabilities (from 0 to 10).

e) The genes from JL03 encoding the proteins identified which also found in the other 3 sequenced serotypes of APP.

f) Genes encode proteins involved in ion-transportation according to COGnitor or Blast at NCBI.
